# Supplementary material for: Intestinal Permeability Study of Clinically Relevant Formulations of Silibinin in Caco-2 Cell Monolayers
Source: Int J Mol Sci. 2019 Mar 31;20(7):1606. doi: 10.3390/ijms20071606 (PMC6480586; doi:10.3390/ijms20071606)
Supplement: Supplementary file 1 [file ijms-20-01606-s001.pdf]

International Journal of
Molecular Sciences

MDPI

Communication

Intestinal permeability study of clinically relevant formulations of silibinin in Caco-2 cell monolayers

Almudena Pérez-Sánchez, Elisabet Cuyàs, Verónica Ruiz-Torres, Luz Agulló-Chazarra, Sara Verdura, Isabel González-Álvarez, Marival Bermejo, Jorge Joven, Vicente Micol, Joaquim Bosch-Barrera, Javier A. Menendez

SUPPLEMENTARY INFORMATION

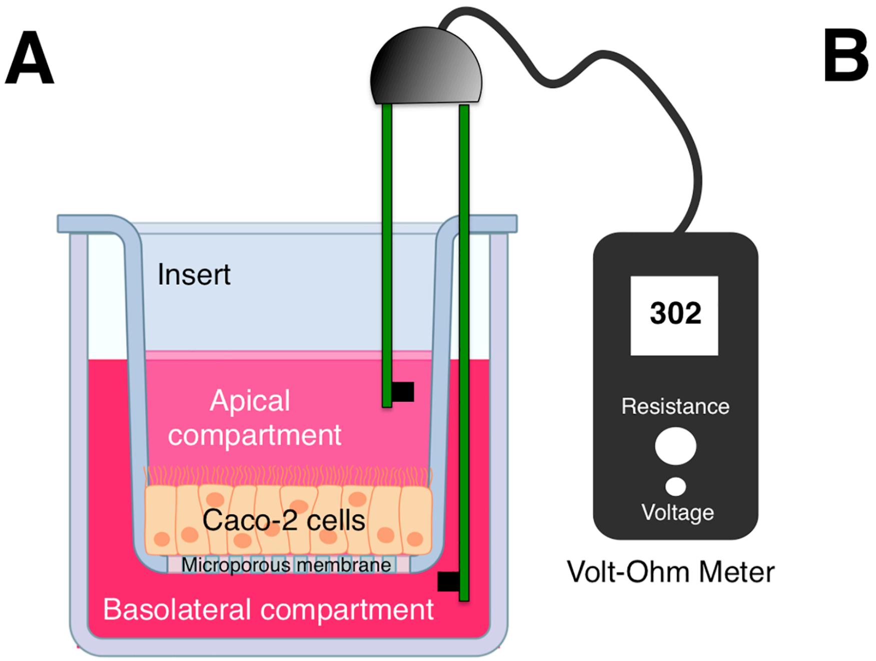

TEER (ohm/cm²)					
Before treatment	After treatment				
**Eurosil85/Euromed**					
**Well #1**	**Well #2**	**Well #3**	**Well #4**	**Well #5**	**Well #6**
370-300	376-304	372-323	370-281	384-285	379-291
368-271	368-304	368-304	368-271	372-271	380-280
**Silibinin-PC/Siliphos®**					
**Well #1**	**Well #2**	**Well #3**	**Well #4**	**Well #5**	**Well #6**
354-315	362-299	370-306	353-282	359-282	378-262
346-311	368-316	369-313	337-299	357-291	366-288
**Silibinin-meglumine**					
**Well #1**	**Well #2**	**Well #3**	**Well #4**	**Well #5**	**Well #6**
396-316	375-310	398-325	393-303	379-312	375-309
367-323	368-319	391-320	367-307	370-307	363-312

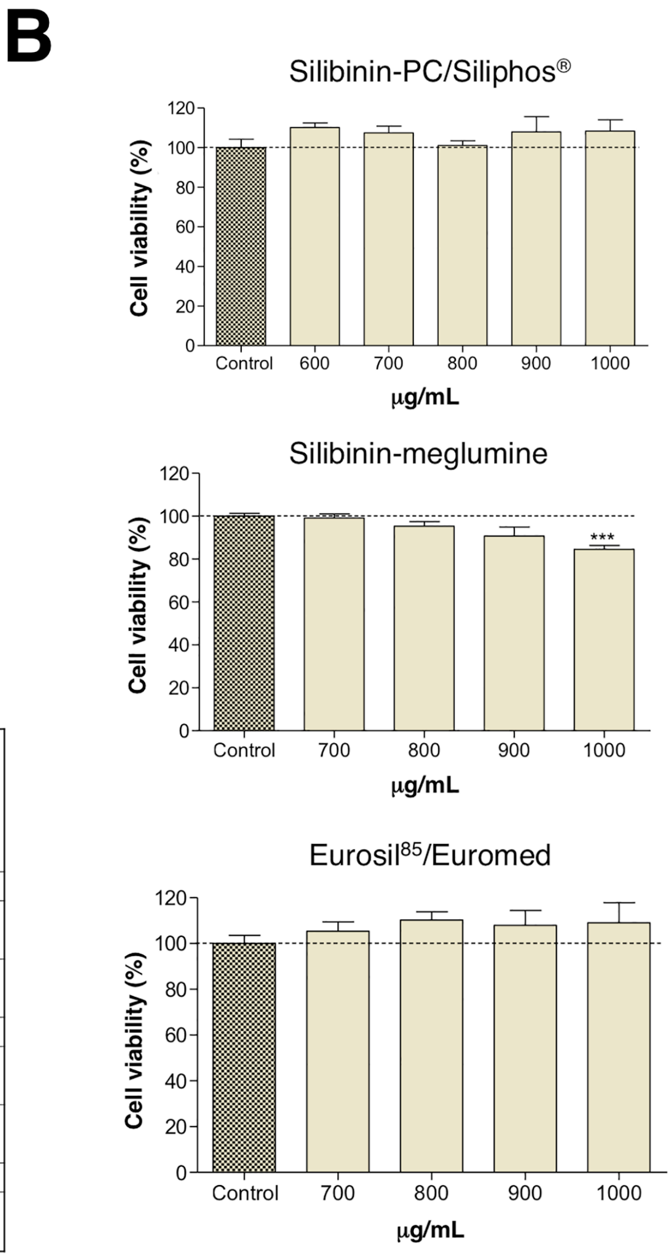

Supplementary Figure S1. A. Changes in the TEER value of Caco-2 monolayers exposed to different silibinin formulations. **B.** Viability of Caco-2 cells after exposure to graded concentrations of silibinin formulations measured by 3-(4,5-Dimethylthiazol-2yl)-2,5-diphenyl-tetrazolium bromide (MTT) assays. Data are presented as percentage of cell viability relative to viability of control cells. Data are expressed as mean ± SEM of 3 independent experiments. Statistical analysis was determined by one-way ANOVA with Tukey's post-hoc test (*** P < 0.001).
